# Diagnosis of *Helicobacter pylori* infection in the elderly using an immunochromatographic assay‐based stool antigen test

**DOI:** 10.1002/mbo3.1102

**Published:** 2020-07-14

**Authors:** Yingjie Han, Wei Dai, Fansen Meng, Xueyang Gan, Miao Liu, Xinli Deng, Yuan Li, Gangshi Wang

**Affiliations:** ^1^ Department of Geriatric Gastroenterology The Second Medical Center Chinese PLA General Hospital Beijing China; ^2^ Office of Information Management The Second Medical Center Chinese PLA General Hospital Beijing China; ^3^ Department of Epidemiology The Second Medical Center Chinese PLA General Hospital Beijing China; ^4^ Department of Laboratory Medicine The Second Medical Center Chinese PLA General Hospital Beijing China

**Keywords:** ^13^C‐urea breath test, elderly, *Helicobacter pylori* stool antigen test, immunochromatographic assay

## Abstract

The diagnostic value of *Helicobacter pylori* stool antigen (HpSA) tests in elderly subjects remains unclear. The objective of this study was to assess the diagnostic accuracy of the immunochromatographic assay‐based HpSA test in a male elderly cohort and identify factors affecting the accuracy. Data for asymptomatic elderly male citizens (≥65 years old) who received health checkups at the Chinese PLA General Hospital between July 2007 and November 2018 were collected. The diagnostic accuracy of the HpSA test was determined using the ^13^C‐urea breath test as a reference standard. Associations between baseline comorbidities and the accuracy of the HpSA test were analyzed. In total, 316 participants were enrolled, including 193 in the pre‐treatment group (77.2 ± 7.8 years old) and 123 in the post‐treatment group (78.7 ± 8.3 years old). The accuracy (91.5%, 91.2%, and 91.9%) and specificity (97.6%, 98.7%, and 96.0%) were high in all participants, pre‐ and post‐treatment groups, respectively. However, sensitivities were only 68.7%, 65.1%, and 75.0%, respectively. In the pre‐treatment group, constipation was associated with decreased sensitivity (*p* = 0.039), while colorectal polyps were associated with increased sensitivity (*p* = 0.010). Multivariate analysis indicated that constipation and colorectal polyps are independent factors for the sensitivity of HpSA in the pre‐treatment group. The immunochromatographic assay‐based HpSA test achieved high accuracy with high specificity but suboptimal sensitivity in the elderly male cohort. Constipation and colorectal polyps were negatively and positively associated with HpSA sensitivity, respectively, in the pre‐treatment group.

## Graphical abstract

The prevalence of *Helicobacter pylori* infection is elevated along with increasing age. Compared with the ^13^C‐urea breath test, the immunochromatographic assay‐based stool antigen test achieves excellent accuracy with high specificity but suboptimal sensitivity in the male elderly population before and after the eradication of *H. pylori*. Caution is needed when applying this test to patients with constipation, which can reduce the sensitivity of the stool antigen test.

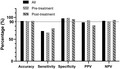

## 1 INTRODUCTION

*Helicobacter pylori* infection is considered an infectious disease, regardless of symptoms and the stage of the disease (Sugano, Tack, & Kuipers, 2015). Along with increasing age, the prevalence of *H*.* pylori* infection is elevated in developing countries (Bardhan, 1997). The reliable diagnosis of *H*.* pylori* infection is of utmost importance for identifying the source of infection, preventing complications related to chronic *H*.* pylori* infection, and monitoring the treatment response after *H*.* pylori* eradication.

Several invasive and noninvasive diagnostic methods for *H*.* pylori* infection are available (Makristathis, Hirschl, & Megraud, 2019). Invasive tests, such as histopathology, *H*.* pylori* culture, rapid urease tests, and modern molecular tests (e.g., real‐time quantitative PCR techniques), require gastroscopy with gastric mucosa biopsies, may need specialized laboratory facilities, and are time‐consuming. Thus, researches have focused on noninvasive methods, such as the urea breath test (UBT), *H*.* pylori* stool antigen (HpSA) test, and serological assays. UBT is capable of identifying active *H*.* pylori* infections and is the most widely studied and preferentially recommended a noninvasive approach for the “test‐and‐treat strategy” (Malfertheiner et al., 2017). The ^13^C‐UBT is the best approach for the detection of *H*.* pylori* infection, with outstanding sensitivity, specificity, and performance (Gisbert & Calvet, 2013; Gisbert & Pajares, 2004a). However, the high price and the need for skilled technical staff and complicated instruments limit the application of UBT in clinical practice. As *H*.* pylori* antibodies may remain positive for several months or longer after the eradication of bacteria, it is difficult to distinguish between current and past infections using serologic tests (Bergey, Marchildon, Peacock, & Megraud, 2003).

The HpSA test detects bacterial antigens and thus can diagnose active infections. It is easy to perform, especially for pediatric and geriatric patients, those with asthma, after gastrectomy, or in the case of achlorhydria, those in which breath test results are unreliable (Yang & Seo, 2008). It is a noninvasive alternative to UBT (Korkmaz, Kesli, & Karabagli, 2013). Previous HpSA tests with poly‐/monoclonal antibodies have shown a sensitivity of 0.83 at a fixed specificity of 0.9 and a ratio of diagnostic odds ratios of 0.88 for the ^13^C‐UBT versus the stool antigen test (Best et al., 2018). The HpSA test can be organized into three groups: immunochromatographic assays (ICA), enzymatic immunoassays (EIA), and immunodot blot assays. *H*.* pylori* stool antigens can be easily and rapidly detected using the ICA‐based HpSA test, with reported sensitivity and specificity values exceeding 90% both before and after *H*.* pylori* treatment (Gatta et al., 2004). There is no significant difference in diagnostic accuracy between ICA‐based tests and EIA‐based tests in children (Yang & Seo, 2008).

The diagnostic value of the HpSA test in elderly patients remains unclear. Only a few reports involving small sample sizes have evaluated HpSA tests in these patients (Inelmen et al., 2004; Kamel et al., 2011; Salles‐Montaudon, Dertheil, & Broutet, 2001, 2002). The objective of this study was to evaluate the sensitivity, specificity, positive (PPV) and negative predictive values (NPV), and diagnostic accuracy of the ICA‐based HpSA test in an elderly male cohort using the ^13^C‐UBT as a reference standard. As elderly individuals often have concurrent chronic diseases, we adjusted their baseline comorbidities to investigate the factors related to the accuracy of ICA‐based HpSA tests in the study population.

## 2 MATERIALS AND METHODS

### 2.1 Participants

Clinical data for elderly male citizens (age ≥65 years) who underwent health checks at the Chinese PLA General Hospital between July 2007 and November 2018 were collected. All participants received the ^13^C‐UBT examination and ICA‐based HpSA test. Stool samples were obtained for the HpSA test, which was performed on the same day or no longer than 1 week before or after the ^13^C‐UBT. Subjects who took antibiotics, proton‐pump inhibitors, H_2_ receptor antagonists, or bismuth within recent 4 weeks of the tests were excluded. Clinical data for concurrent drug use and chronic diseases that may affect the accuracy of tests, such as atrophic gastritis, constipation, colon diverticulum, and diabetes mellitus, were recorded. The history of anti‐*H*.* pylori* treatment (triple or quadruple regimens) was also collected. Subjects with no history of anti‐*H*.* pylori* treatment before ^13^C‐UBT and HpSA tests were regarded as the pre‐treatment group. Those who were tested after anti‐*H*.* pylori* treatment were assigned to the post‐treatment group, irrespective of the frequency and/or outcome of the treatment. Also, gastric and colorectal polyps diagnosed by endoscopy within 3 years of HpSA detection were collected for the baseline comorbidity assessment. Patients with prior gastrointestinal cancer, overt gastrointestinal bleeding, and a history of gastrectomy were excluded. Subjects with intermediate HpSA results were also eliminated from the analysis.

This research was approved by the Ethics Committee of the Chinese PLA General Hospital.

### 2.2 ^13^C‐UBT detection

*Helicobacter pylori* infection was determined via the ^13^C‐UBT. After fasting for over 8 hr, each subject drank a solution containing 75 mg of ^13^C‐urea in 70 ml of water. Breath samples were collected before and 30 min after the ingurgitation of the water. Then, ^13^C‐enrichment was detected using a ^13^C‐breath test instrument (Fischer Analysen Instrumente GmbH). The results were defined as positive when the delta over baseline (DOB) was >4‰, calculated as the surplus of the isotopic ratio over the baseline isotopic ratio.

### 2.3 HpSA test

Fresh fecal samples were used for the analysis. A one‐step chromatographic immunoassay, CerTest *H*.* pylori* Blister Test (CerTest Biotec S.L.), was applied for the analysis, following the manufacturer's instructions. Based on the condition of the control line and sample line, samples were categorized as positive, negative, or intermediate. All intermediate data were excluded from the final analysis.

### 2.4 Statistical analyses

Statistical analyses were performed using Statistical Package for Social Sciences version 25.0 (SPSS, Chicago, IL, USA). Sensitivity, specificity, PPV, and NPV with 95% confidence intervals (CI) were calculated by standard methods using ^13^C‐UBT as the reference standard. Continuous variables are expressed as means ± standard deviation (*SD*). Categorical variables are expressed as *n* (%). The chi‐square test was used to detect differences within categorical variables. A univariate analysis was performed for all variables, including age, comorbidities, and medications, via chi‐square tests. A multivariate analysis was performed to determine independent factors for diagnostic efficiency using a binary logistic regression model. A *p*‐value of less than 0.05 (two‐sided) was regarded as statistically significant.

## 3 RESULTS

### 3.1 Demographic characteristics of participants

A total of 316 participants who underwent both ^13^C‐UBT and HpSA tests were enrolled. Among them, 193 subjects were assigned to the pre‐treatment group and 123 subjects were assigned to the post‐treatment group. The mean ages of all participants and those in the pre‐treatment and post‐treatment groups were 77.8 ± 8.0 years, 77.2 ± 7.8 years, and 78.7 ± 8.3 years, respectively. The positive rate for ^13^C‐UBT was 21.1% and that for HpSA was 16.5% (Table 1 and Table A1 in the Appendix A). Comorbidities in each group, such as atrophic gastritis, constipation, colorectal polyps, diabetes, hyperlipidemia, hypertension, and dementia, are listed in Table 1. A total of 72 (22.8%) participants suffered from constipation and 131 (41.5%) participants had colorectal polyps. Medications administered to the participants are summarized in Table A2 in the Appendix A.

**TABLE 1:** Demographic characteristics of all participants

Characteristics	All (*N* = 316)	Pre‐treatment (*N* = 193)	Post‐treatment (*N* = 123)
Mean ± *SD*			
Age (years)	77.8 ± 8.0	77.2 ± 7.8	78.7 ± 8.3
*N* (%)			
^13^C‐UBT‐positive	67 (21.1)	43 (22.3)	24 (19.5)
HpSA test‐positive	52 (16.5)	30 (15.5)	22 (17.9)
Atrophic gastritis	189 (59.8)	115 (59.6)	74 (60.2)
GERD	53 (16.8)	29 (15.0)	24 (19.5)
Constipation	72 (22.8)	44 (22.8)	28 (22.8)
Colon diverticulum	34 (10.8)	14 (7.3)	20 (16.3)
Gastric polyps	27 (8.5)	19 (9.8)	8 (6.5)
Colorectal polyps	131 (41.5)	76 (39.4)	55 (44.7)
History of intestinal surgery	8 (2.5)	3 (1.6)	5 (4.1)
Diabetes mellitus	109 (34.5)	70 (36.3)	39 (31.7)
Hyperlipidemia	49 (15.5)	31 (16.1)	18 (14.6)
Coronary heart disease	166 (52.5)	95 (49.2)	71 (57.7)
Hypertension	189 (59.8)	113 (58.5)	76 (61.8)
COPD	77 (24.4)	37 (19.2)	40 (32.5)
Dementia	10 (3.2)	8 (4.1)	2 (1.6)
Post‐cerebral infarction	64 (20.3)	40 (20.7)	24 (19.5)
Median (IQR)			
Days between ^13^C‐UBT and HpSA test (days)	2 (1–4)	2 (1–4)	2 (1–4)

Abbreviations: COPD, chronic obstructive pulmonary disease; GERD, gastroesophageal reflux disease; HpSA, *Helicobacter pylori* stool antigen; IQR, interquartile range; *N*, number; *SD*, standard deviation; UBT, urea breath test.

### 3.2 Diagnostic efficacy of HpSA test

**FIGURE 1:**
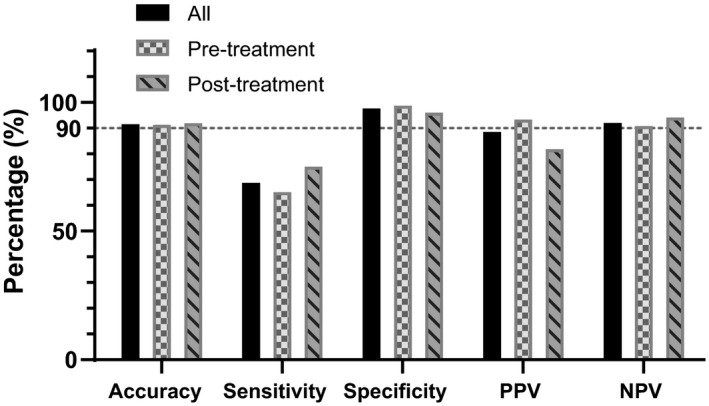
Diagnostic efficacy of the HpSA test for *Helicobacter pylori* detection. ^13^C‐UBT served as a reference standard. HpSA, *Helicobacter pylori* stool antigen; NPV, negative predictive value; PPV, positive predictive value

The performance of the HpSA test using ^13^C‐UBT as a reference standard was analyzed. The median time between ^13^C‐UBT and HpSA tests was 2 days (interquartile range 1–4 days). As shown in Figure 1 and Table A3 in the Appendix A, the accuracy of the HpSA test was 91.5% (95% CI: 87.8%–94.3%) for all participants, 91.2% (95% CI: 86.3%–94.8%) in the pre‐treatment group, and 91.9% (95% CI: 85.6%–96.0%) in the post‐treatment group. The specificities were 97.6% (95% CI: 94.6%–99.0%), 98.7% (95% CI: 94.8%–99.8%), and 96.0% (95% CI: 89.4%–98.7%) in these groups. However, the sensitivities were only 68.7% (95% CI: 56.0%–79.1%), 65.1% (95% CI: 49.0%–78.5%), and 75.0% (95% CI: 52.9%–89.4%) in these groups. The PPV was 88.5% (95% CI: 75.9%–95.2%) in all subjects, 93.3% (95% CI: 76.5%–98.8%) in the pre‐treatment group, and 81.8% (95% CI: 59.0%–94.0%) in the post‐treatment group, and the NPV was 92.0% (95% CI: 87.9%–94.9%), 90.8% (95% CI: 85.0%–94.6%), and 94.1% (95% CI: 87.0%–97.6%) in these groups. Intermediate results of HpSA tests and their potential influence on the diagnostic accuracy are listed in Tables A4 and A5 in the Appendix A.

### 3.3 Factors associated with HpSA sensitivity

We further investigated factors (comorbidities) that affect the sensitivity of the HpSA test. Univariate analysis indicated that the sensitivity of the HpSA test was significantly higher for participants over 78 years old than for younger participants (54.8% vs. 80.6%, *p* = 0.024), significantly lower in those with constipation than in those without (76.5% vs. 43.8%, *p* = 0.014), and higher in those with colorectal polyps than in those without (56.8% vs. 83.3%, *p* = 0.020). In the pre‐treatment group, the sensitivity of the HpSA test was significantly lower in participants with constipation than in those without constipation (76.7% vs. 38.5%, *p* = 0.039). The sensitivity was significantly higher in participants with colorectal polyps than those without (45.0% vs. 82.6%, *p* = 0.010). Differences in these parameters were not observed in the post‐treatment group (Table 2). Other comorbidities, such as colon diverticulum, a history of bowel surgery, diabetes, and hyperlipidemia, were not significantly correlated with HpSA sensitivity in this cohort. No medications were significantly associated with HpSA sensitivity in all groups (Table A6 in Appendix A).

**TABLE 2:** Comorbidities affecting HpSA test sensitivity

Factors	Subgroups	All		Pre‐treatment		Post‐treatment	
		Sensitivity	*p*	Sensitivity	*p*	Sensitivity	*p*
Age (years)	<78	54.8%	0.024*	54.5%	0.137	55.6%	0.224
	≥78	80.6%		76.2%		86.7%	
Atrophic gastritis	−	58.6%	0.122	55.0%	0.194	66.7%	0.808
	+	76.3%		73.9%		80.0%	
GERD	−	67.8%	0.995	64.1%	1.000	75.0%	1.000
	+	75.0%		75.0%		75.0%	
Constipation	−	76.5%	0.014*	76.7%	0.039*	76.2%	0.722
	+	43.8%		38.5%		66.7%	
Colon diverticulum	−	68.9%	1.000	64.3%	1.000	78.9%	0.772
	+	66.7%		100.0%		60.0%	
Gastric polyps	−	68.3%	1.000	65.0%	1.000	73.9%	1.000
	+	75.0%		66.7%		100.0%	
Colorectal polyps	−	56.8%	0.020*	45.0%	0.010 *	70.6%	0.795
	+	83.3%		82.6%		85.7%	
History of intestinal surgery	−	68.2%	1.000	65.1%	NULL	73.9%	1.000
	+	100.0%		NULL		100.0%	
Diabetes mellitus	−	69.2%	0.905	68.2%	0.666	70.6%	0.795
	+	67.9%		61.9%		85.7%	
Hyperlipidemia	−	73.2%	0.145	71.4%	0.160	76.2%	1.000
	+	45.5%		37.5%		66.7%	
Coronary heart disease	−	69.0%	0.962	73.7%	0.294	60.0%	0.339
	+	68.4%		58.3%		85.7%	
Hypertension	−	64.3%	0.513	66.7%	0.856	60.0%	0.339
	+	71.8%		64.0%		85.7%	
COPD	−	63.8%	0.192	62.5%	0.805	66.7%	0.465
	+	80.0%		72.7%		88.9%	
Dementia	−	68.8%	1.000	65.0%	1.000	75.0%	NULL
	+	66.7%		66.7%		NULL	
Post‐cerebral infarction	−	65.3%	0.329	61.3%	0.625	72.2%	1.000
	+	77.8%		75.0%		83.3%	

Abbreviations: COPD, chronic obstructive pulmonary disease; GERD, gastroesophageal reflux disease; HpSA, *Helicobacter pylori* stool antigen.

* *p* < 0.05.

To identify the most important covariate for HpSA sensitivity, various factors including age, constipation, and colorectal polyps were subjected to multivariate regression analysis. Both constipation and colorectal polyps were independent factors for the sensitivity of the HpSA test in all participants and the pre‐treatment group. All summary statistics are summarized in Table 3.

**TABLE 3:** OR for factors affecting HpSA test sensitivity

Groups	Factors	Regression coefficient	*SE* for regression coefficient	*p*	OR (95% CI)
All	Constipation	−2.01	0.74	0.007*	0.134 (0.032–0.570)
	Colorectal polyps	1.87	0.72	0.009*	6.492 (1.591–26.482)
Pre‐treatment	Constipation	−2.16	0.89	0.016*	0.115 (0.020–0.666)
	Colorectal polyps	2.21	0.87	0.011*	9.095 (1.656–49.955)

Abbreviations: CI, confidence interval; HpSA, *Helicobacter pylori* stool antigen; OR, odds ratio; *SE*, standard error.

* *p* < 0.05.

A subgroup analysis showed that the accuracy of the HpSA test in patients with constipation was lower than that in patients without constipation (94.3% vs. 81.9%) for all participants (Table A7 in Appendix A). A similar tendency was found in the pre‐treatment group (95.3% vs. 77.3%) (Table A8 in Appendix A). The accuracy of the HpSA test in patients with colorectal polyp was higher than that in non‐colorectal polyp counterparts (88.1% vs. 96.2%) for all participants (Table A7 in the Appendix A) and the pre‐treatment group (88.9% vs. 94.7%) (Table A8 in the Appendix A).

## 4 DISCUSSION

Monoclonal HpSA test is considered a precise, noninvasive method for the diagnosis of *H*.* pylori *infection and proof of *H*.* pylori* eradication (Gisbert, de la Morena, & Abraira, 2006; McNicholl et al., 2019). It is approved by the FDA of the United States and is recommended by clinical guidelines as a substitute for invasive diagnostic methods (Malfertheiner et al., 2017). The HpSA test only requires fresh stool samples and therefore is particularly useful in relatively old patients and especially in individuals who are not able to expire air completely or who cannot swallow. Furthermore, the HpSA test has higher precision than UBT in patients with a history of subtotal gastrectomy (Costa et al., 2001). Data for the sensitivity and specificity of ICA‐based HpSA in asymptomatic older subjects are lacking. Using the ^13^C‐UBT as a reference standard, our results showed that ICA‐based HpSA has a very high specificity (all: 97.6%, pre‐treatment: 98.7%, post‐treatment: 96.0%) but a relatively low sensitivity (all: 68.7%, pre‐treatment: 65.1%, post‐treatment: 75.0%), with an accuracy of greater than 91% in each group. The results were comparable to those of a previous study of hospitalized elderly patients (≥65 years old), in which the sensitivity and specificity of the Premier Platinum HpSA test (EIA‐based HpSA) in untreated patients were 76% and 93%, respectively (Inelmen, Gasparini, & Sergi, 2005). Similar results were reported in a study involving 122 elderly hospitalized patients (Inelmen et al., 2004). These data indicated that the sensitivity of the HpSA test could be inferior to those of other tests in elderly individuals.

Some studies have suggested that *H*.* pylori* shedding decreases with an increase in the chronicity of infection (Haggerty, Perry, Sanchez, Perez‐Perez, & Parsonnet, 2005), which is commonly observed in the elderly population. The prolonged passage of bacteria into the colon owing to chronic constipation may also lead to the degradation of *H*.* pylori* antigens (Monteiro, Gras, Vidal, Cabrita, & Mégraud, 2001), thereby decreasing the accuracy of the HpSA test. Consistent with these previous results, our univariate and multivariate analyses revealed a significantly lower sensitivity of the HpSA test in subjects with constipation than in those without constipation in the pre‐treatment group. However, we found no correlation between HpSA sensitivity and drugs with the potential to affect passage through the colon, such as opioid analgesics.

We noted that colorectal polyps were closely related to the sensitivity of the HpSA test (88.9% in participants with colorectal polyps vs. 50.0% in participants without colorectal polyps). *Helicobacter pylori* infection has been identified as an independent risk factor for colorectal polyps and colonic adenomas, especially in cases of advanced or multiple lesions (Dong, Guo, & Yang, 2019; Nam et al., 2017); we detected a correlation between *H*.* pylori* infection and colorectal polyps in the pre‐treatment group but not in the post‐treatment group or the whole cohort (Table A9 in the Appendix A). We did not find an association between polyps and constipation in the pre‐treatment group (Table A10 in Appendix A). Large prospective studies are needed for further investigation of the association between colorectal polyps and HpSA sensitivity in the elderly.

Low sensitivity of the polyclonal HpSA test in the post‐treatment setting has been reported (Gisbert & Pajares, 2004b). However, there is evidence that the HpSA test using monoclonal antibodies shows superior sensitivity to those of tests using polyclonal antibodies, particularly in the post‐treatment setting (Gisbert et al., 2006). We achieved an accuracy rate exceeding 91% in both pre‐ and post‐treatment groups by using ICA, consistent with previous reports (Vaira et al., 2000). Our stringent inclusion criteria may explain the high accuracy obtained in both the pre‐ and post‐treatment groups. In this study, factors with the potential to affect the accuracy of ^13^C‐UBT or HpSA, such as proton‐pump inhibitors, antibiotics, bismuth therapy (Calvet et al., 2002; Gisbert & Pajares, 2001; Grino et al., 2003; Inelmen et al., 2005), a history of gastrectomy, and overt gastrointestinal bleeding, were excluded. Cases with “indeterminate results” were also excluded. Furthermore, the ^13^C‐UBT and HpSA tests were performed on the same day or with an interval of no longer than 7 days to minimize the error caused by variation in detection times. Finally, we recruited only asymptomatic elderly subjects who underwent the tests for health checkup purposes to minimize the influence of active and/or severe diseases.

There were several limitations to this study. First, ^13^C‐UBT, believed to be an ideal noninvasive assay, was chosen as the only reference standard (Best et al., 2018). According to the literature, the false‐negative rate of ^13^C‐UBT could be elevated in elderly individuals (Salles‐Montaudon et al., 2001). A combination of invasive tests, such as histological or culture data, would be a more effective reference standard. Also, the sample size was relatively small and was limited to male subjects. Sample size calculation was performed based on the following settings: the prevalence of *H*.* pylori* infection (around 25%) in the cohort, previously reported sensitivity (76%) and specificity (93%) values for the HpSA test in the elderly (Inelmen et al., 2005), and a two‐sided α level of 0.05. We noticed that only the whole study cohort matched the sample size requirement. Although no study has reported a gender difference in noninvasive detection efficiency, further studies of both male and female subjects will broaden our knowledge in this field. Moreover, participants were in a relatively higher‐than‐average socioeconomic status, as evidenced by their utilization of a regular health check with a low prevalence (22.3%) of *H*.* pylori* infection, compared with a reported prevalence in Beijing, China, in the general population of as high as 47.0% (Hooi et al., 2017). Hence, the generalizability of the results of this study to the whole elderly population should be performed with caution. Lastly, we cannot confirm the causality between the parameters identified, such as colorectal polyps, and HpSA sensitivity, as this is an observational study.

## 5 CONCLUSIONS

In an observational study of an elderly male cohort, we revealed that HpSA achieves high accuracy and specificity but suboptimal sensitivity in both pre‐ and post‐treatment groups when using ^13^C‐UBT as a reference standard. Our findings show that ICA‐based HpSA is accurate for the diagnosis of *H*.* pylori* infection in the elderly. We found that comorbidities, such as constipation and colorectal polyps, can affect the sensitivity of HpSA. Owing to the lower sensitivity of the HpSA test, caution should be taken when applying this test to elderly patients with constipation.

## CONFLICT OF INTEREST

None declared.

## AUTHOR CONTRIBUTIONS

**Yingjie Han:** Data curation (lead); formal analysis (lead); project administration (supporting); writing – original draft (lead); writing – review & editing (supporting). **Wei Dai:** Investigation (equal). **Fansen Meng:** Investigation (equal). **Xueyang Gan:** Investigation (equal). **Miao Liu:** Formal analysis (supporting). **Xinli Deng:** Investigation (supporting); resources (supporting). **Yuan Li:** Investigation (supporting); resources (supporting). **Gangshi Wang:** Conceptualization (lead); Data curation (supporting); investigation (supporting); project administration (lead); resources (lead); supervision (lead); writing – review & editing (lead).

## ETHICS STATEMENT

This retrospective study complies with the Declaration of Helsinki and was following standards of the ethical committee of the Chinese PLA General Hospital. Because data collection was based on retrospective searches of electronic medical records and patient identities were not disclosed, consent was not available and was not required according to the ethics committee of Chinese PLA General Hospital.

## Data Availability

All primary data obtained in this study excluding information related to participant identities are shown in Supplementary Table A11 available in the Zenodo repository (https://doi.org/10.5281/zenodo.3871777).

## Appendix A

**TABLE A1:** Summary of ^13^C‐UBT and HpSA test results

Groups	HpSA test	^13^C‐UBT		
		+	−	Total
All participants	+	46	6	52
	−	21	243	264
	Total	67	249	316
Pre‐treatment	+	28	2	30
	−	15	148	163
	Total	43	150	193
Post‐treatment	+	18	4	22
	−	6	95	101
	Total	24	99	123

Abbreviations: HpSA, *Helicobacter pylori* stool antigen; UBT, urea breath test.

**TABLE A2:** Summary of medications administered to study participants

Medications	All (*N* = 316)	Pre‐treatment (*N* = 193)	Post‐treatment (*N* = 123)
*N* (%)			
Prokinetic agents	18 (5.7)	11 (5.7)	7 (5.7)
Opioid analgesics	44 (13.9)	22 (11.4)	22 (17.9)
Prebiotics or probiotics	37 (11.7)	24 (12.4)	13 (10.6)
Digestive enzyme	22 (7)	12 (6.2)	10 (8.1)
Antiplatelet agents	40 (12.7)	28 (14.5)	12 (9.8)
Antihypertensive agents	189 (59.8)	113 (58.5)	76 (61.8)
Lipid‐lowering agents	57 (18)	44 (22.8)	13 (10.6)
α‐glucosidase inhibitor	14 (4.4)	12 (6.2)	2 (1.6)

**TABLE A3:** Diagnostic efficacy of the HpSA test for *Helicobacter pylori* detection^a^

Groups	Accuracy (95% CI)	Sensitivity (95% CI)	Specificity (95% CI)	PPV (95% CI)	NPV (95% CI)
All participants	91.5% (87.8%–94.3%)	68.7% (56.0%–79.1%)	97.6% (94.6%–99.0%)	88.5% (75.9%–95.2%)	92.0% (87.9%–94.9%)
Pre‐treatment	91.2% (86.3%–94.8%)	65.1% (49.0%–78.5%)	98.7% (94.8%–99.8%)	93.3% (76.5%–98.8%)	90.8% (85.0%–94.6%)
Post‐treatment	91.9% (85.6%–96.0%)	75.0% (52.9%–89.4%)	96.0% (89.4%–98.7%)	81.8% (59.0%–94.0%)	94.1% (87.0%–97.6%)

Abbreviations: CI, confidence interval; HpSA, *Helicobacter pylori* stool antigen; NPV, negative predictive value; PPV, positive predictive value; UBT, urea breath test.

^a^ Excluding intermediate results for the HpSA test.

**TABLE A4:** Diagnostic efficacy of the HpSA test for *Helicobacter pylori* detection^a^

Groups	Accuracy (95% CI)	Sensitivity (95% CI)	Specificity (95% CI)	PPV (95% CI)	NPV (95% CI)
All participants	87.7% (87.7%–87.8%)	70.4% (59.8%–81.0%)	92.4% (89.2%–95.6%)	71.4% (60.8%–82.0%)	92.0% (88.8%–95.3%)
Pre‐treatment	87.3% (87.2%–87.4%)	67.4% (53.8%–80.9%)	93.1% (89.1%–97.0%)	73.8% (60.5%–87.1%)	90.8% (86.4%–95.2%)
Post‐treatment	88.4% (88.2%–88.5%)	76.0% (59.3%–92.7%)	91.3% (85.9%–96.7%)	67.9% (50.6%–85.2%)	94.1% (89.4%–98.7%)

Abbreviations: CI, confidence interval; HpSA, *Helicobacter pylori* stool antigen; NPV, negative predictive value; PPV, positive predictive value; UBT, urea breath test.

^a^ Treating intermediate results for the HpSA test as positive results.

**TABLE A5:** Diagnostic efficacy of the HpSA test for *Helicobacter pylori* detection^a^

Groups	Accuracy (95% CI)	Sensitivity (95% CI)	Specificity (95% CI)	PPV (95% CI)	NPV (95% CI)
All participants	90.7% (90.7%–90.8%)	64.8% (53.7%–75.9%)	97.7% (95.9%–99.5%)	88.5% (79.8%–97.1%)	91.1% (87.8%–94.5%)
Pre‐treatment	90.2% (90.2%–90.3%)	60.9% (46.8%–75.0%)	98.7% (97.0%–100.0%)	93.3% (84.4%–100.0%)	89.7% (85.2%–94.2%)
Post‐treatment	91.5% (91.4%–91.6%)	72.0% (54.4%–89.6%)	96.2% (92.5%–99.8%)	81.8% (65.7%–97.9%)	93.5% (88.8%–98.1%)

Abbreviations: CI, confidence interval; HpSA, *Helicobacter pylori* stool antigen; NPV, negative predictive value; PPV, positive predictive value; UBT, urea breath test.

^a^ Treating intermediate results of the HpSA test as negative results.

**TABLE A6:** Medications affecting the sensitivity of the HpSA test

Factors	Subgroups	All		Pre‐treatment		Post‐treatment	
		Sensitivity	*p*	Sensitivity	*p*	Sensitivity	*p*
Prokinetic agents	−	68.9%	1.000	63.2%	0.807	78.3%	0.250
	+	66.7%		80.0%		0.0%	
Opioid analgesics	−	66.1%	0.413	63.2%	0.807	71.4%	0.546
	+	87.5%		80.0%		100.0%	
Prebiotics or probiotics	−	67.8%	0.995	62.2%	0.584	77.3%	0.446
	+	75.0%		83.3%		50.0%	
Digestive enzyme	−	69.8%	0.784	65.9%	1.000	77.3%	0.446
	+	50.0%		50.0%		50.0%	
Antiplatelet agents	−	69.0%	1.000	63.9%	1.000	77.3%	0.446
	+	66.7%		71.4%		50.0%	
Antihypertensive agents	−	64.3%	0.513	66.7%	0.856	60.0%	0.339
	+	71.8%		64.0%		85.7%	
Lipid‐lowering agents	−	68.5%	1.000	64.5%	1.000	73.9%	1.000
	+	69.2%		66.7%		100.0%	
α‐glucosidase inhibitor	−	70.5%	0.568	67.6%	0.707	75.0%	NULL
	+	50.0%		50.0%		NULL	

Abbreviation: HpSA, *Helicobacter pylori* stool antigen.

**TABLE A7:** Performance of the HpSA test in all participants^a^

Subgroup	HpSA test	^13^C‐UBT			Accuracy (95% CI)	Sensitivity (95% CI)	Specificity (95% CI)	PPV (95% CI)	NPV (95% CI)
		+	−	Total					
Non‐constipation	+	39	2	41	94.3% (94.2%–94.3%)	76.5% (64.8%–88.1%)	99.0% (97.5%–100%)	95.1% (88.5%–100%)	94.1% (90.8%–97.3%)
	−	12	191	203					
	Total	51	193	244					
Constipation	+	7	4	11	81.9% (81.5%–82.3%)	43.8% (19.4%–68.1%)	92.9% (86.1%–99.6%)	63.6% (35.2%–92.1%)	85.2% (76.3%–94.1%)
	−	9	52	61					
	Total	16	56	72					
Non‐colorectal polyps	+	21	6	27	88.1% (88.0%–88.2%)	56.8% (40.8%–72.7%)	95.9% (92.8%–99.1%)	77.8% (62.1%–93.5%)	89.9% (85.2%–94.6%)
	−	16	142	158					
	Total	37	148	185					
Colorectal polyps	+	25	0	25	96.2% (96.1%–96.2%)	83.3% (70.0%–96.7%)	100.0% (100%–100%)	100.0% (100%–100%)	95.3% (91.2%–99.3%)
	−	5	101	106					
	Total	30	101	131					

Abbreviations: CI, confidence interval; HpSA, *Helicobacter pylori* stool antigen; NPV, negative predictive value; PPV, positive predictive value; UBT, urea breath test.

^a^ ^13^C‐UBT served as the gold standard.

**TABLE A8:** Performance of HpSA test in the pre‐treatment subgroup^a^

Subgroup	HpSA test	^13^C‐UBT			Accuracy (95% CI)	Sensitivity (95% CI)	Specificity (95% CI)	PPV (95% CI)	NPV (95% CI)
		+	−	Total					
Non‐constipation	+	23	0	23	95.3% (90.6%–98.1%)	76.7% (57.3%–89.4%)	100.0% (96.1%–100.0%)	100.0% (82.2%–100.0%)	94.4% (88.5%–97.5%)
	−	7	119	126					
	Total	30	119	149					
Constipation	+	5	2	7	77.3% (62.2%–88.5%)	38.5% (15.1%–98.9%)	93.5% (77.2%–98.9%)	71.4% (30.3%–98.9%)	78.4% (61.3%–98.9%)
	−	8	29	37					
	Total	13	31	44					
Non‐colorectal polyps	+	9	2	11	88.9% (81.7%–93.9%)	45.0% (23.8%–68.0%)	97.9% (92.0%–99.6%)	81.8% (47.8%–96.8%)	89.6% (81.8%–94.5%)
	−	11	95	106					
	Total	20	97	117					
Colorectal polyps	+	19	0	19	94.7% (87.1%–98.5%)	82.6% (60.5%–94.3%)	100.0% (91.6%–100.0%)	100.0% (79.1%–100.0%)	93.0% (82.2%–97.7%)
	−	4	53	57					
	Total	23	53	76					

Abbreviations: CI, confidence interval; HpSA, *Helicobacter pylori* stool antigen; NPV, negative predictive value; PPV, positive predictive value; UBT, urea breath test.

^a^ ^13^C‐UBT served as the reference standard.

**TABLE A9:** Relationship between *Helicobacter pylori* infection and colorectal polyps

Groups	Subgroups	^13^C‐UBT‐negative	^13^C‐UBT‐positive	χ^2^	*p*
All participants	Non‐colorectal polyps	148	37	0.386	0.534
	Colorectal polyps	101	30		
Pre‐treatment	Non‐colorectal polyps	97	20	4.614	0.032*
	Colorectal polyps	53	23		
Post‐treatment	Non‐colorectal polyps	51	17	2.916	0.088
	Colorectal polyps	48	7		

Abbreviation: ^13^C‐UBT, ^13^C‐urea breath test.

* *p* < 0.05.

**TABLE A10:** Relationship between constipation and colorectal polyps in *Helicobacter pylori*‐infected participants

Groups	Subgroups	Colorectal polyps *N* (%)	χ^2^	*p*
All participants	Non‐constipation	21 (41.2)	1.119	0.290
	Constipation	9 (56.3)		
Pre‐treatment	Non‐constipation	16 (53.3)	0.001	0.975
	Constipation	7 (53.8)		

Abbreviation: UBT, urea breath test.
